# Surgical outcome of hepatocellular carcinoma patients with biliary tumor thrombi

**DOI:** 10.1186/1477-7819-9-2

**Published:** 2011-01-08

**Authors:** Wenyu Shao, Chengjun Sui, Zhenyu Liu, Jiamei Yang, Yanming Zhou

**Affiliations:** 1 Department of Liver Surgery, The First Affiliated Hospital of Nanjing Medical University, Nanjing, PR China; 2 Department of Hepato-Biliary-Pancreato-Vascular Surgery, the First Affiliated Hospital of Xiamen University, Xiamen, PR China; 3 Department of Special Treatment and Liver transplantation, Eastern Hepatobiliary Surgery Hospital, Second Military Medical University, Shanghai, PR China

## Abstract

**Background:**

To investigate the surgical outcome of hepatocellular carcinoma (HCC) patients with biliary tumor thrombi (BTT).

**Methods:**

Surgical outcome of 27 HCC patients with BTT (group I) were compared with randomly selected HCC patients without BTT (group II; n = 270).

**Results:**

One patient in group I died of hepatic failure within 30 days after resection. The 1-, 3- and 5-year cumulative survival rates of group I were 70.3%, 25.9%, and 7.4%, respectively; these were significantly lower than those of group II (90.6%, 54.0%, and 37.7%) (*P <*0.001). The rates of early recurrence (≤ 1 year) after resection were significantly higher in group I than group II (70.3% vs. 34.8%) (*P *< 0.001).

**Conclusion:**

HCC patients with BTT had a worse prognosis after resection than those without BTT. Resection should be considered for these tumors given the lack of effective alternative therapies.

## Background

Hepatocellular carcinoma (HCC), one of the most common malignancies worldwide, can frequently invade the portal vein and cause portal vein tumor thrombus. By contrast, biliary tumor thrombi (BTT) is rare, and the incidence ranging from 0.53% to 12.9% in autopsy and surgical specimens [1-4]. HCC patients with BTT have a poor prognosis. Nonsurgical treatment modalities, such as transcatheter arterial chemoembolization (TACE), internal biliary stenting, radiotherapy, often has disappointing outcomes. Surgical treatment is the only way that possibly cures the patients. However, the role of hepatic resection in such patients is controversial [2,5-7].

The aim of the present study was to investigate the surgical outcome of HCC patients with BTT by comparing with those without BTT.

## Methods

### Patients

From January 2000 to December 2006, 1246 patients underwent hepatic resections for HCC, at the Department of Liver Surgery, the First Affiliated Hospital of Nanjing Medical University, and Department of Special Treatment and Liver transplantation in Eastern Hepatobiliary Surgery Hospital of Second Military Medical University. Among them, 27 patients were found having BTT (group I). According to classification proposed by Esaki *et al*. [8], three patients (11.1%) had microscopic BTT and 24 patients (88.9%) had macroscopic BTT. For comparison of surgical results, 270 patients were randomly chosen from the remaining 1,219 HCC patients without BTT and matched 10:1 with group I by age, sex, concomitant liver background, and resection margins (group II).

Routine imaging studies included chest radiography, abdominal ultrasonography, and abdominal computed tomography. Endoscopic retrograde cholangiopancreatography or magnetic resonance cholangiopancreatography were employed to evaluate the extension of a BTT. Evaluation of liver function included serum biochemistry, prothrombin time. Serum hepatitis B surface antigen (HBsAg) and hepatitis C antibody were used as the positive markers of chronic viral hepatitis infection. HCC was diagnosed by at least two radiologic imaging showing characteristic features of HCC; or one radiologic imaging showing characteristic features of HCC associated with alpha-fetoprotein (AFP) > 400 ng/ml; or cytologic/histologic evidence [9]. Operative procedures were determined by preoperative diagnosis of location of the primary tumors and the extension of BTT.

Tumor size was measured directly in the surgical specimen by pathology examination. A tumor satellite is defined as any daughter tumor < 3 cm in size lying within a 3-cm zone from the dominant tumor [10]. The histological differentiation of HCC was graded according to the criteria of Edmondson and Steiner (G1, well differentiated; G2, moderately differentiated; G3, poorly differentiated; G4, undifferentiated) [11]. Macrovascular invasion was defined as gross invasion of the right or left main branches of the portal vein or the hepatic veins [12]. Microvascular invasion indicated the presence of clusters of cancer cells floating in the vascular space line by endothelial cells on histopathologic examination [13]. The diagnosis of liver cirrhosis was based on the histology.

Perioperative deaths were defined as either within 30 days of surgery or occurring in hospital.

This retrospective study was approved by the ethics committee of the two hospitals.

### Follow-up

After discharge, patients were followed-up every one month by AFP analysis and ultrasound or computed tomography at least every three months at our outpatient clinic, especially during the first two years. Patients who developed recurrence were treated with re-resection whenever possible, or by TACE, percutaneous ethanol injection, or radiofrequency ablation as appropriate. According to point of recurrences time from the date of hepatectomy, recurrences were classified into early (≤ 1 year) and late (> 1 year) recurrences [14].

### Statistical analysis

Categorical and continuous variables were compared with chi-square test and *t *test, respectively. Overall survival rates were estimated with the Kaplan-Meier product-limit method and compared by log-rank test. All statistical analyses were performed using SPSS for Windows (version 11.0; SPSS Institute, Chicago, IL, USA). *P *< 0.05 was considered statistically significant.

## Results

### Patients features

The clinical data are presented in Table 1. Compared with groups II, group I patients had a higher incidence of carbohydrate antigen19-9 (CA19-9) > 37 U/ml, higher serum levels of total bilirubin, alanine aminotransferase (ALT), aspartate aminotransferase (AST), γ-glutamyl transpeptidase (GGT), and alkaline phosphatase (ALP) (*P <*0.001). There were no differences in age, sex, serology for viral hepatitis, and serum albumin levels among the two groups. Patients with serum AFP levels greater than 400 ng/ml were found more frequently in the group I than in the groups II, but this result did not reach statistical significance (*P *= 0.058).

**Table 1:** Clinical features between two groups

Variables	Group I (n = 27)	Group II (n = 270)	*P value*
Age (years))	47.1 ± 10.5	48.0 ±11.3	0.272
Sex (Male/Female)	24/3	232/38	0.670
HBsAg-positive	26 (96.7%)	254 (94.1%)	0.636
Anti-HCV-positive	0	2 (1.2%)	0.654
Serum AFP (> 400 ng/ml)	16 (59.3%)	109 (40.3%)	0.058
Serum CA19-9 (> 37 U/ml)	13 (48.1%)	24 (8.8%)	< 0.001
Serum total bilirubin (umol/L)	116.4 ± 135.4	14.5 ± 7.8	< 0.001
Serum ALT (IU/L)	132.2 ± 107.9	59.6 ± 53.0	< 0.001
Serum AST (IU/L)	95.58 ± 51.5	60.1 ± 53.8	< 0.001
Serum albumin (g/L)	40.4 ± 4.92	41.8 ± 5.6	0.326
Serum GGT (IU/L)	583.1 ± 372.4	122.9 ± 134.1	< 0.001
Serum ALP (IU/L)	305.4 ± 148.0	132.2 ± 83.1	< 0.001

HBsAg: hepatitis B surface antigen. HCV: hepatitis C virus. AFP: alpha-fetoprotein.

CA19-9: carbohydrate antigen19-9. BTT: biliary tumor thrombi.

ALT: alanine aminotransferase. AST: aspartate aminotransferase. GGT: γ-glutamyl transpeptidase.

ALP: alkaline phosphatase.

### Pathologic features

The pathologic features of HCC patients with or without BTT underwent hepatic resection are shown in Table 2. The incidence of tumor size ≤ 5 cm tended to be higher in group I, but this result did not reach statistical significance (*P = *0.091). The incidence of microscopic vascular invasion, tumor capsule absence, and high Edmondson- Steiner grade in group I was significantly higher than that in group II (*P <*0.05). There were no significant differences between the two groups with respect to cirrhosis, resection margins, macroscopic vascular invasion, lymph node metastasis, and tumor satellites.

**Table 2:** Comparison of pathologic features between group I and II

Variables	Group I (n = 27)	Group II (n = 270)	*P value*
Cirrhosis	18 (66.7%)	161 (59.6%)	0.476
Tumor size ≤ 5 cm	17 (62.9%)	124 (41.4%)	0.091
Resection margins ≤ 1 cm	3 (11.1%)	32 (11.8%)	0.909
Macrovascular invasion	3 (11.1%)	26 (9.6%)	0.805
Microvascular invasion	17 (62.9%)	116 (43.0%)	0.046
Lymph node metastasis	1 (3.7%)	8 (2.9%)	0.830
Tumor satellites	5 (18.5%)	42 (15.6%)	0.688
Tumor capsule absence	21 (77.7%)	142 (52.6%)	0.012
Edmonson-Steiner grade			
1-2	1 (3.7%)	56 (20.1%)	0.036
3-4	26 (96.3%)	214 (79.9%)	

### Surgical results

The surgical procedures used in group I patients included right anterior resection (n = 1), right posterior resection (n = 2), right hepatectomy (n = 4), left hepatectomy (n = 8), left hepatectomy with caudate lobectomy (n = 1), left lateral recection (n = 3), left medial recection (n = 2), and partial resection (n = 6). The BTT were removed by the choledochotomy in 18 cases, from the cut end of the bile duct after liver resection in five cases, and *en bloc *removal with the primary tumor in three cases. Extrahepatic bile duct resection and biliaryenteric anastomosis was performed in one case.

**Figure 1:**
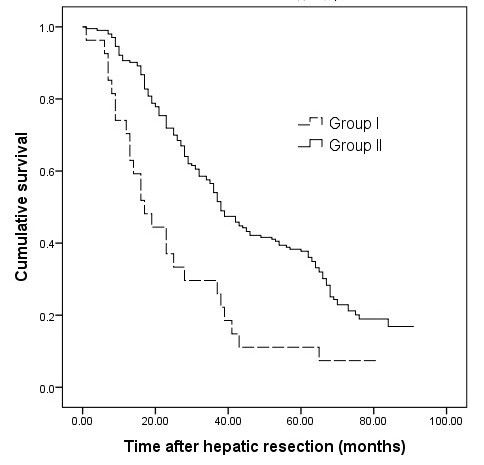
**Overall survival of patients in two groups**. The overall survival of group I was lower than group II (*P *< .001).

One patient in group I died of hepatic failure within 30 days after resection. There was no perioperative mortality in group II. The 1-, 3- and 5-year cumulative survival rates of group I were 70.3%, 25.9%, and 7.4%, respectively; these were significantly lower than those of group II (90.6%, 54.0%, and 37.7%) (*P <*0.001) (Figure 1).

During the median follow-up period of 39 months postresection (range 1 to 104 months), 25 of patients (92.5%) in group I and 218 patients (80.7%) in group II experienced intrahepatic recurrence. The rates of early recurrence (≤1 year) after resection were significantly higher in group I than group II (70.3% vs. 34.8%) (*P <*0.001).

Seven patients in group I recurrenced with BTT again and had obstructive jaundice. One patient received re-resection, six were palliated with endoscopic stents.

## Discussion

In 1949, Mallory *et al*. [15] described a single case of HCC invading the gall bladder and obstructing extrahepatic bile ducts. In 1975 Lin *et al*. [16] described eight patients and classified icteric type hepatoma. Since then, there are several reports concerning HCC with BDT have been published [1-7]. The outcome in patients who received palliative treatment was poor, with a mean survival time of less than 5 months [17-19]. Surgical treatment is the only way that possibly cures the patients [19,20]. However, the resectability rate of such tumors was very low in early reports, ranged only from 2% to 13.2% [21]. In recent years, with progress in imaging diagnosis and preoperative management, the number of resectable cases were increased remarkably [4-7,21].

HCC invades into biliary system through one of the following three mechanisms: (1) the tumor may grow continuously in a distal fashion, filling the entire extrahepatic biliary system with a solid cast of tumor; (2) a fragment of necrotic tumor may separate from the proximal intraductal growth, migrating to the distal common bile duct and causing obstruction; or (3) hemorrhage from the tumor may partially or completely fill the biliary tree with blood clots [18]. Generally, the tumor thrombus was not adherent to the bile duct wall so it could be removed easily. Tumor thrombi rarely invade the walls of the large bile ducts around the hepatic hilus. Therefore, liver resection of the involved hepatic segments with thrombectomy through a choledochotomy is a rational technique for curative resection [2]. The indications for extrahepatic bile duct resection were macroscopic tumor invasion of the large bile ducts around the hepatic hilus [2].

Venous invasion is a well-established prognostic indicator of HCC [22,23]. By contrast, whether BTT have a significant impact on the prognosis of HCC remain controversial. Shiomi *et al*. [2] reported that the 3- and 5-year survival rates were 47% and 28%, respectively, in 17 patients with BTT, similar to those achieved in 115 patients without BTT. Satoh *et al*. [5] also found that there were no significant differences in survival between patients with BTT and those without BTT. These data suggest that BTT in HCC patients might have lower aggressive potential and be less important as a prognostic factor. Paradoxically, Yeh *et al*. [6] in an analysis of 17 patients who underwent resection, reported that the overall survival was worse in patients with BTT, compared with those without BTT. In another recent study, Ikenaga *et al*. [7] reported that the median survival time of HCC patients with BTT after surgery was significantly shorter than that of those without thrombi (11.4 vs. 56.1 months, *P *= 0.002).

Currently, there are two surgical staging systems, which were developed based on the analysis of patients who received hepatic resection: one from the Liver Cancer Study Group of Japan (LCSGJ) and another from the American Joint Committee on Cancer (AJCC)/International Union Against Cancer (UICC). In LCSGJ system, presence of BTT was considered as an indicator of "advanced stage" of HCC. A study from Japan reported that the prognostic stratification ability of the LCSGJ staging system is superior to that of the AJCC/UICC staging system and that the BTT is the strongest prognostic factor for HCC [24]. This idea is supported by the results in our present study showing that the resection of HCC with BTT results in a significantly worse survival outcome compared with hepatic resection of HCC without BTT.

In current study, the incidence of microscopic vascular invasion [13,23], tumor capsule absence [25,26], high Edmondson- Steiner grade [22,27,28], and high level of GGT [29], unfavorable clinical prognostic factors, in group I were significantly higher than the respective factors in group II. Therefore, these factors may account for the poor prognosis in patients with BTT, at least in part. In addition, the incidences of CA19-9 > 37 U/ml, elevation of total bilirubin, ALT, AST, and ALP in group I were significantly higher than those in group II, which may be related to obstructive jaundice caused by BTT.

Previous studies have indicated that the HCC patients with BTT had smaller tumors and a higher percentage of a tumor size ≤ 5 cm than those without BTT [6]. Similarly, in this study, the incidence of tumor size ≤ 5 cm tended to be higher in group I, despite this result did not reach statistical significance due to the small number of patients. Although the exact mechanism remains unknown, some authors have suggested that a BTT in HCC tends to grow faster than the primary tumor itself [4].

Tumor recurrence remains the major cause of death after resection for HCC [30,31]. Despite similar treatments, the prognosis for patients with early recurrence was worse than that of patients with late recurrence [14]. Qin *et al*. [4] reported that 14 patients with BTT (14/28, 50.0%) were found intrahepatic HCC recurrence within 1 year after operation. Ikenaga *et al*. [7] reported that 53% of patients suffered recurrences in the remnant liver within 3 months after surgery. In current study, patients with BTT developed early recurrence after resection more frequently.

We found seven patients in group I recurrenced with BTT again. Recurrence in the liver remnant could results from either intrahepatic metastasis from the primary tumor or multicentric occurrence. Intrahepatic metastasis is an important mechanism of early intrahepatic recurrence after resection of HCC. Spreading via the portal vein is considered the main route of intrahepatic metastasis [14,32]. In current study, 17 patients (62.9%) in the group I had microscopic vascular invasion. However, nine of the other ten cases without venous invasion also developed early recurrence. Similar results had been published previously by Ikenaga *et al*. [7], who found that two patients with BTT without portal vein invasion suffered multiple recurrences in the remnant liver. The authors speculate that HCC invasion biliary system may be another route of intrahepatic metastasis.

Esaki *et al*. [8] reported that HCC patients with macroscopic BTT had a better postoperative survival than patients with microscopic BTT. But Ikenaga *et al*. [7] found that the prognosis was similar between two groups. In current study, statistical analysis was limited by the too small population with microscopic BTT.

The experience with orthotopic liver transplantation (OLT) in patients with BTT is limited. There were only five cases reported in the English literatures [21,33]. Of these patients, one died 20 months after OLT due to multiple intrahepatic recurrences, one developed carcinoma recurrence at the lower end of the common bile duct 27 months after OLT. The other three patients were alive without evidence of recurrence during the follow-up period (17.6 to 28.1 months). These results suggest that OLT may be a treatment option for HCC with BDT in selected cases.

## Conclusions

HCC patients with BTT had a worse prognosis after resection than those without BTT. Resection should be considered for these tumors given the lack of effective alternative therapies. Further studies are needed for understanding of molecular biology of BTT may yield therapeutic tools that can improve the prognosis of this subset of patients.

## Competing interests

The authors declare that they have no competing interests.

## Authors' contributions

WS participated in the design and coordination of the study, carried out the critical appraisal of studies and wrote the manuscript. CS, ZL, and YZ developed the literature search, carried out the extraction of data, assisted in the critical appraisal of included studies and assisted in writing up. WS and CS carried out the statistical analysis of studies. JY and YZ interpreted data, corrected and approve the manuscript. All authors read and approved the final manuscript.

## Consent

Written informed consent was obtained from the patients for publication of this case series. A copy of the written consent is available for review by the Editor-in-Chief of this journal.
